# Causal relationship between systemic lupus erythematosus and coronary artery disease: Insights from a meta-analysis and Mendelian randomization

**DOI:** 10.1097/MD.0000000000048748

**Published:** 2026-05-15

**Authors:** Ping Yuan, Li Fu, Min Ge, Yan Fan

**Affiliations:** a Department of Geriatrics, The Third Hospital of Changsha, Changsha, China.

**Keywords:** atherosclerosis, coronary artery disease, Mendelian randomization, meta-analysis, systemic lupus erythematosus

## Abstract

Observational research has produced varied results concerning the association between systemic lupus erythematosus (SLE) and coronary artery disease (CAD). This study employed a meta-analysis of cohort studies alongside a 2-sample Mendelian randomization (MR) method to investigate the causal influence of SLE on CAD risk. A comprehensive literature search was executed across PubMed, Web of Science, Embase, and the Cochrane Library, covering all pertinent cohort studies from their inception to August 14, 2024. The data regarding the causal association between SLE and CAD were synthesized using relative risk (RR), with results presented within a 95% confidence interval (CI). For MR analysis, data were derived from genome-wide association studies (GWAS) focusing on SLE and CAD in European and East Asian populations, respectively. The primary MR analysis employed the inverse-variance weighted (IVW) method, with the weighted median method and MR-Egger regression serving as complementary approaches. This meta-analysis included 13 cohort studies with a total of 2517,781 participants. The combined findings indicated that compared to the general or healthy population, individuals with SLE had a higher risk of developing CAD in the total population (RR [95% CI] = 2.355 [1.924–2.883], 95% prediction interval [PI]: 1.199–4.628). This association was also observed among European (RR [95% CI] = 2.028 [1.310–3.138], 95% PI: 0.539–7.631), East Asian (RR [95% CI] = 2.628 [1.698–4.067], 95% PI: 0.014–487.335), and North American (RR [95% CI] = 2.711 [2.379–3.089], 95% PI: 2.193–3.352) groups. However, MR analysis utilizing the IVW method did not identify a genetically predicted causal relationship between SLE and CAD in either European or East Asian populations (all *P* > .05). Sensitivity analyses indicated an absence of heterogeneity or horizontal pleiotropy in the MR analysis. Findings from our meta-analysis indicated that SLE is associated with an elevated risk of CAD. Despite the lack of genetic evidence for a direct causal relationship between SLE and CAD, clinicians should remain vigilant and take proactive measures in monitoring CAD among SLE patients, considering the possible indirect effects of SLE on CAD risk.

## 1. Introduction

Systemic lupus erythematosus (SLE) is a persistent autoimmune disorder capable of inducing significant dysfunction in the kidneys, central nervous system, and heart.^[1]^ Globally, SLE affects between 20 and 150 individuals per 100,000, with women being 9 times more likely to be affected than men.^[2]^ The disease is linked to a markedly increased mortality burden, approximately double that of the general population,^[3,4]^ with late-stage deaths predominantly caused by infections and atherosclerotic heart disease.^[1]^ Advances in survival rates over recent decades are largely due to early detection and aggressive immunosuppressive treatment at the onset of the disease, which have helped prevent end-organ damage.^[5]^ As a result, cardiovascular disease (CVD) has become the predominant cause of death among SLE patients.^[5]^ Coronary artery disease (CAD), the most prevalent type of CVD, accounts for 30% of deaths and one-third to one-half of all CVD cases.^[6,7]^ Large-scale epidemiological research has highlighted CAD as a major concern for individuals suffering from SLE.^[8,9]^

Currently, research examining the incidence and risk of CAD in individuals with SLE has yielded inconsistent findings.^[10–12]^ In addition, a recent pooled analysis of observational studies reported no significant elevation in CAD risk among SLE patients.^[13]^ Conversely, another combined analysis indicated a notably higher risk.^[14]^ The disparity in outcomes from these studies, coupled with the reliance on observational research methods-such as case-control and cross-sectional designs-raises concerns about potential reverse causality. To address these limitations, we performed a meta-analysis focusing on eligible cohort studies to derive more precise causal inferences regarding CAD risk in SLE patients. Nonetheless, accurately determining CAD risk in this population remains challenging due to confounding factors inherent in traditional epidemiological research.

Mendelian randomization (MR) is an approach employing genetic variation to investigate causal relationships between exposures and outcomes.^[15]^ This method relies on the principle of random genetic distribution, which renders its findings resistant to confounding factors and reverse causation.^[16]^ Since genotypes are determined at conception and precede the development of outcomes, they remain unaffected by numerous confounders, including environmental influences, making MR a powerful tool for studying causal associations.^[17]^ In this research, we initially performed a meta-analysis using cohort study data. Subsequently, MR was employed to validate and corroborate the meta-analysis findings. These results may provide new insights into the association between SLE and CAD.

## 2. Methods

### 2.1. Meta-analysis

#### 2.1.1. Study design and search strategy

This meta-analysis adheres to the Preferred Reporting Items for Systematic Reviews and Meta-Analyses guidelines.^[18]^ The study protocol has been registered with PROSPERO, bearing the ID: CRD42024580883. Comprehensive searches were conducted in PubMed, the Cochrane Library, Embase, and Web of Science to assess the causal relationship between SLE and the risk of CAD from their inception up to August 14, 2024. The search terms included: (“systemic lupus erythematosus,” “lupus erythematosus, systemic,” “lupus erythematosus disseminatus,” “libman sacks disease,” “libman-sacks disease,” “disease, libman-sacks”) AND (“coronary artery disease,” “disease, coronary artery,” “artery disease, coronary,” “coronary arteriosclerosis,” “coronary arterioscleroses,” “coronary atherosclerosis,” “coronary atheroscleroses”). The search was conducted without restrictions on date or language. Detailed search methodologies for each database were provided in Supplementary Flies 1. Furthermore, the references cited in pertinent review articles were examined to determine their relevance.

#### 2.1.2. Inclusion and exclusion criteria

**Figure 1.**
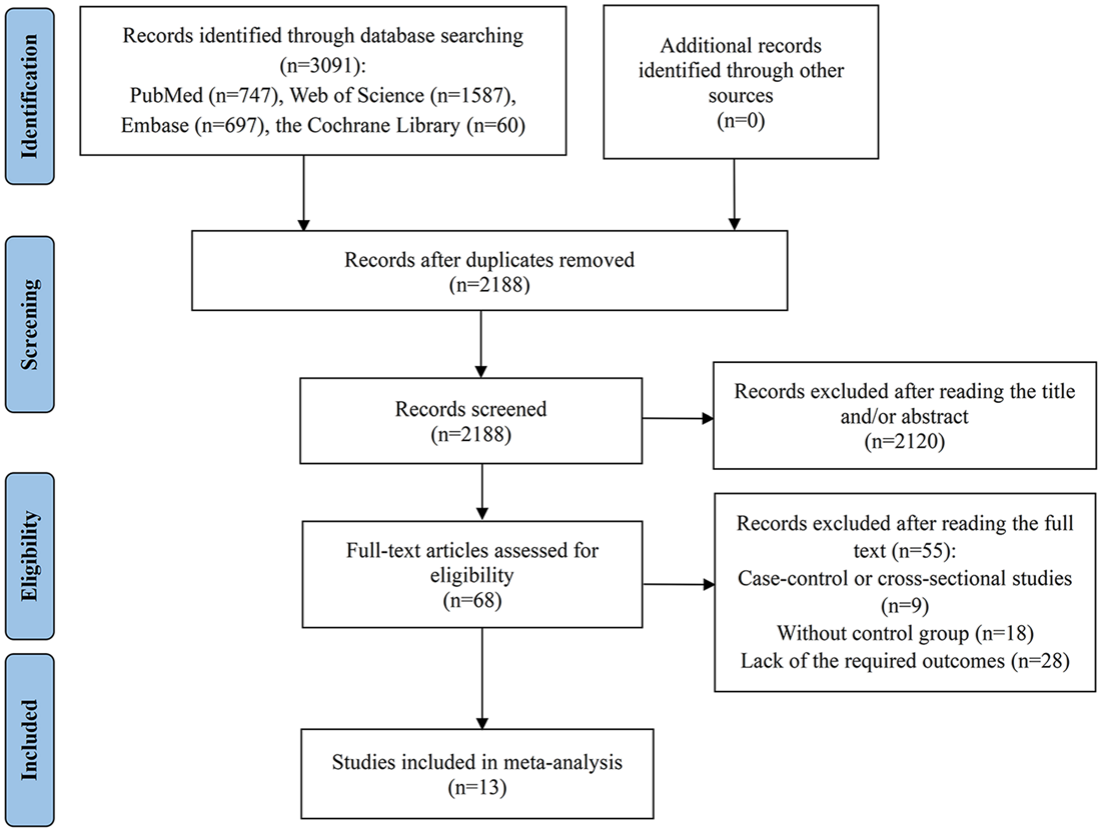
Flow diagram of the process of study selection in meta-analysis.

The inclusion criteria for the studies were as follows: only cohort studies, encompassing both retrospective and prospective designs, were eligible; investigations focused on assessing the risk of CAD in patients with SLE; the research provided hazard ratios (HRs), relative risks (RRs), or odds ratios (ORs), along with their 95% confidence intervals (CIs), comparing CAD risk in SLE patients with that in the general population or healthy controls; and SLE diagnosis was confirmed using recognized international criteria, such as the International Classification of Diseases codes and the American College of Rheumatology guidelines. The exclusion criteria included: case-control or cross-sectional studies were omitted; studies lacking control groups were excluded; research not providing required outcome data was disregarded; non-human studies were not considered; and case reports, conference abstracts, and review articles were excluded from the analysis. Figure 1 illustrated the Preferred Reporting Items for Systematic Reviews and Meta-Analyses flowchart detailing the literature that was included and excluded from the analysis.

#### 2.1.3. Data extraction and quality assessment

Two researchers independently employed a pre-designed data extraction form to gather information from the included studies. The information gathered from the selected studies encompassed fundamental study characteristics, such as the first author’s name, publication year, geographic location, study design, participant age, and sample size. Additionally, the diagnostic criteria for SLE and CAD, definitions of CAD, and adjusted confounding factors were recorded. Any inconsistencies encountered during the extraction process were resolved through consensus, with the involvement of a third reviewer. To evaluate the quality of the included cohort studies, the Newcastle-Ottawa Scale (NOS) was applied,^[19]^ which considers subject selection, group comparability, and the assessment of outcome. Each study was categorized as low (0–3 points), moderate (4–6 points), or high (7–9 points) quality.^[20]^ Two authors performed the bias risk assessments, resolving any differences through discussion.

#### 2.1.4. Statistical analysis

The study primarily employed pooled RR and corresponding 95% CI as the main summary metrics. OR and HR were incorporated as estimates to combine with RR.^[14]^ Heterogeneity among studies was assessed using the I^2^ statistic, Cochran Q test, and a 95% prediction interval (PI).^[21,22]^ A random-effects model was selected when I^2^ exceeded 50% or the *P*-value was <.10; otherwise, a fixed-effects model was chosen.^[23]^ Subgroup analyses were performed to investigate the causal link between SLE and CAD across various ethnic groups. Sensitivity analysis, which involved sequentially excluding each study, was conducted to assess the robustness of the meta-analysis findings. Publication bias was examined through Begg and Egger tests, complemented by funnel plot assessments.^[24,25]^ Data analysis and visualization were carried out using R software 4.3.1 and STATA 12.0. All *p*-values were 2-tailed, with a significance threshold set at *P* < .05.

### 2.2. Mendelian randomization analysis

#### 2.2.1. Research design and data sources

**Figure 2.**
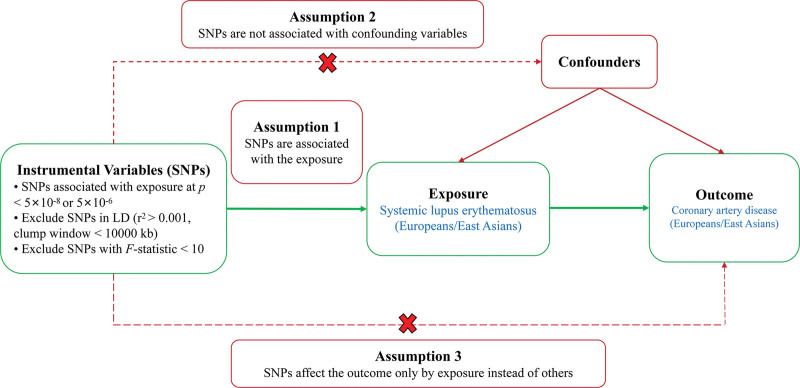
Study design diagram and 3 assumptions of Mendelian randomization. SNPs, single nucleotide polymorphisms; LD, linkage disequilibrium.

A 2-sample MR analysis was conducted to explore the causal relationship between SLE and CAD. To ensure the validity of the MR analysis, 3 key assumptions had to be met: the IVs must have a strong correlation with the exposure variable; the IVs must be independent of any confounders that could influence the exposure-outcome relationship; and the IVs should affect the outcome solely through the exposure pathway (Fig. 2).^[26]^ This research adhered to the STROBE-MR guidelines.^[27]^ We utilized large-scale, publicly available genome-wide association studies (GWAS) summary data for MR analyses in both European and East Asian populations. The European SLE data were obtained from the IEU Open GWAS project (https://gwas.mrcieu.ac.uk/), specifically under the GWAS ID ebi-a-GCST90018917. CAD genetic data at the summary level were sourced from a GWAS using the UK Biobank, encompassing up to 352,063 participants.^[28]^ For the East Asian cohort, SLE GWAS data were obtained from the Biobank Japan project, the largest biobank focused on the East Asian population.^[29]^ Additionally, CAD GWAS data (ID: bbj-a-159) were sourced from the IEU project, based on the GWAS conducted by Biobank Japan. Table 1 presented the summary statistics of the genetic variants associated with these traits. Ethics approval and written informed consent were not required for MR analysis, as the GWAS data utilized were publicly accessible and had previously been approved by the appropriate ethical review boards in the original studies.

**Table 1:** The GWAS data source details in our Mendelian randomization study.

Population	Phenotype	Cases	Controls	Sample size	nSNP	Data source
European	SLE	647	482,264	482,911	24,198,877	IEU Open GWAS
	CAD	352,063 British ancestry individuals			11,027,870	GWAS catalog
East Asian	SLE	317	175,937	176,254	12,457,681	GWAS catalog
	CAD	29,319	183,134	212,453	8881,048	IEU Open GWAS

CAD = coronary artery disease, SLE = systemic lupus erythematosus.

#### 2.2.2. Instrumental variable selection

Single nucleotide polymorphisms (SNPs) were employed as instrumental variables (IVs) in this analysis. For the European cohort, where SLE was the exposure variable, we applied a significance threshold of *P* < 5 × 10^-8^. However, for the East Asian cohort, this threshold was relaxed to *P* < 5 × 10^-6^ to ensure a sufficient number of SNPs were available for conducting heterogeneity and pleiotropy tests. To maintain independence, we selected SNPs separated by more than 10,000 kb and with a low likelihood of linkage disequilibrium (r^2^ < 0.001). The *F*-statistic was employed to evaluate potential IV bias, with an *F* value >10 chosen to prevent weak IVs from affecting causal inference.^[30]^ SNPs showing an association with the exposure and a direct connection to outcome, with a *P*-value less than 5 × 10^-8^, were excluded. Furthermore, SNPs absent from the outcome GWAS dataset, those with allele inconsistencies between exposure and outcome, and palindromic SNPs that could introduce bias were also removed.

#### 2.2.3. Statistical analysis

To elucidate the causal relationship between SLE and CAD, we employed 2-sample MR analyses utilizing several methodologies, including inverse variance weighting (IVW), the weighted median approach, and MR-Egger regression. As IVW yields the most precise causal estimates when all IVs are valid, we employed the fixed-effects IVW as our primary method. In cases where heterogeneity was detected, the random-effects IVW was utilized.^[31]^ Cochran Q test was conducted to evaluate the consistency of the effect estimates from the selected IVs on the exposure, with *P* < .05 indicating significant heterogeneity. MR-Egger regression was utilized to identify and adjust for directional pleiotropy, while the weighted median method was employed to provide robust causal estimates even if up to 50% of the IVs were invalid. The integration of these methods strengthens the reliability of our causal conclusions. Additionally, sensitivity analyses were conducted to assess the robustness of the associations. Horizontal pleiotropy was assessed using the MR-Egger intercept, where a *P*-value exceeding 0.05 suggested the absence of pleiotropy. Subsequently, the MR pleiotropy residual sum and outlier (MR-PRESSO) test was performed to identify and correct horizontal pleiotropic outliers, ensuring accurate results by eliminating any outliers.^[32]^ To assess the influence of individual SNPs on the overall statistical findings, a leave-one-out analysis was performed. This approach elucidated the specific contributions of each SNP to the the final results. To further confirm the robustness of our findings, funnel plot analysis was undertaken. All analyses were conducted using the “TwoSampleMR,” “MR-PRESSO,” and “MendelianRandomization” packages in R software 4.3.1. A 2-sided *P*-value of <.05 was considered statistically significant.

## 3. Results

### 3.1. Meta-analysis results

#### 3.1.1. Study selection, characteristics and quality assessment

The initial database search identified 3091 records. After excluding 3023 studies due to duplication or failure to meet the inclusion criteria, 68 studies were selected for full-text review. Ultimately, 13 cohort studies were included in the meta-analysis.^[10–12,33–42]^ Figure 1 illustrated the detailed selection process and the rationale behind the exclusion of the remaining 55 studies. The included studies consisted of 10 retrospective cohort studies and 3 prospective cohort studies, encompassing 82,205 cases of SLE and 2435,576 controls at baseline. Geographically, 5 studies were conducted in Europe, 3 in East Asia, and 5 in North America. Detailed characteristics of the included studies were provided in Table 2. Moreover, every study included received a high-quality rating, achieving scores exceeding 7 based on the NOS quality assessment tool. The specifics of the quality assessment for these studies were provided in Table S1.

**Table 2:** The basic characteristics of all included studies in this meta-analysis.

Author (Year)	Region	Study design	SLE criteria	Sample size (SLE cases/Controls)	Age (SLE cases/Controls), years	Definition of CAD	Diagnosis of CAD	Follow-up duration, years	Adjustments	NOS score
Barbhaiya (2020)	USA	RCS	ICD-9	40,212/160,848	40.34 ± 12.14/40.34 ± 12.14	Non-fatal acute MI	ICD-9	1.86 ± 1.11/1.73 ± 1.14	Age, sex, race/ethnicity, region of residence, year and zip code-level socioeconomic status, charlson score, number of medications, cardiac comorbidities at index date	7
Baena-Díez (2018)	Spain	RCS	ICD-10	664/952,489	NR/52 ± 13	Incident MI or angina	ICD-10	2007.1.1-2012.12.31	Age, sex, smoking status, total cholesterol, high density lipoprotein cholesterol, systolic blood pressure, diastolic blood pressure, statins, hypertensive drugs and 3 categories of exposure to antirheumatic-specific treatments: disease-modifying antirheumatic drugs, other anti-inflammatory drugs, no exposure	8
Kaul (2013)	USA	RCS	ICD-9	86/258	Median (IQR): 49 (40–58)/70 (63–78)	CAD	ICD-9	4.3 (IQR 1.9–8.0)	Age, sex, history of MI, and hyperlipidemia	8
Lim (2018)	Korea	RCS	ICD-10	18,575/92,875	20–65+	MI	ICD-10	8	Age, sex, low income, diabetes mellitus, hypertension, and dyslipidemia	8
Aviña-Zubieta (2017)	Canada	RCS	ICD-9 or ICD-10	4863/49,316	48.5 ± 16.1/48.6 ± 16.1	MI	ICD-9 or ICD-10	1996.1–2010.12	Age, sex, entry time, no. of outpatient visits	8
Hak (2009)	USA	PCS	ACR criteria (≥ 4 criteria)	148/119,184	52.6 ± 8.6/ 56.2 ± 7.2	Coronary heart disease	Criteria of the WHO	28	Age in years, history of hypertension, diabetes, and hypercholesterolemia, parental history of coronary heart disease before the age of 60, body mass index, physical exercise, smoking status, alcohol consumption, menopausal status and use of hormone replacement therapy, aspirin, non-steroidal anti-inflammatory drugs and oral corticosteroids and race	8
Hermansen (2017)	Denmark	RCS	ICD-10	1644/8220	NR	MI	ICD-8 or ICD-10	Median (IQR): 7.8 (3.3–12.7)	Sex, age, income and Charlson Comorbidity Index score	8
Bengtsson (2012)	Sweden	RCS	SLICC/ACR (≥ 4)	258/516	51.2 ± 14.9/48.4 ± 12.4	MI	ICD-10	7	Age, previous MI and AP with coronary artery intervention	8
Tornvall (2021)	Sweden	RCS	ICD-8 or ICD-9	4192/41,892	55 ± 18/55 ± 18	Acute MI	ICD-8 or ICD-9	20	Age, gender, hypertension, diabetes mellitus, renal disease, hyperlipidemia, obesity, COPD, liver cirrhosis, and mental disorders	8
Goldberg (2009)	Canada	PCS	ACR classification criteria ≥ 4 or ACR = 3 with a typical biopsy lesion of SLE	241/237	44.2 ± 12.2/44.5 ± 14.4	MI and/or angina pectoris	Telephone interview and chart review	7.2 ± 2.3	Age, total triglycerides	8
Kravvariti (2018)	Greece	PCS	SLEDAI-2K and physician global assessment	101/85	44 ± 12/44 ± 13	Subclinical atherosclerosis	Ultrasound methodology	3	Risks conferred by traditional factors using Systemic Coronary Risk Evaluation (SCORE)	7
Lai (2022)	China	RCS	ICD-9	10,014/1000,000	39.4 ± 15.3/43.1 ± 16.3	CAD	ICD-9	5.3 (Mean)	Age, sex, DM, HTN, dyslipidaemia, renal failure, atherosclerosis, steroids, antidiabetics, diuretics, beta-blockers, calcium channel blocker, lipid lowing agents, aspirin, non-steroid anti-inflammatory drugs	8
Lin (2014)	China	RCS	ICD-9	1207/9656	> 20	Acute MI	ICD-9	2000-2008.12.31	Age, sex, urbanization, low income, coexisting medical conditions and medication use	8

ACR = American College of Rheumatology, AP = angina pectoris, CAD = coronary artery disease, COPD = chronic obstructive pulmonary disease, DM = diabetes mellitus, HTN = hypertension, ICD = International Classification of Diseases, IQR = interquartile range, MI = myocardial infarction, NR = not reported, PCS = prospective cohort study, RCS = retrospective cohort study, SLE = systemic lupus erythematosus, SLEDAI-2K = SLE disease activity index 2000, SLICC = Systemic Lupus International Collaborating Clinics.

#### 3.1.2. Meta-analysis of the causal association between SLE and CAD

**Figure 3.**
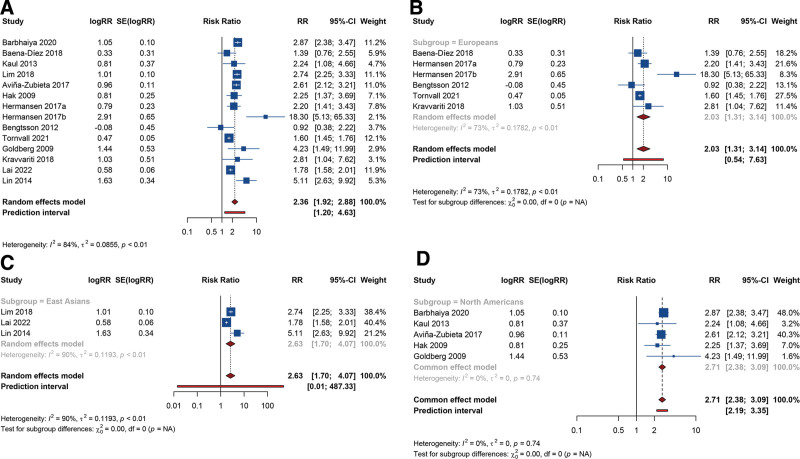
Meta-analysis of the causal association between systemic lupus erythematosus and coronary artery disease. (A) Overall analysis; (B) Subgroup = European; (C)Subgroup = East Asian; (D) Subgroup = North American.

Hermansen et al assessed the risk of CAD in SLE patients, differentiating between those with and without lupus nephritis.^[37]^ Consequently, their data were treated as 2 separate studies in our pooled analysis. Utilizing a random-effects model (I^2^ = 83.9%, Tau^2^ = 0.0855), the overall analysis revealed that compared with the general population or healthy controls, SLE significantly increased the risk of CAD in the total population (RR [95% CI] = 2.355 [1.924–2.883], 95% PI: 1.199–4.628) (Fig. 3A). Subgroup analyses, stratified by the ethnicity of the participants, indicated a significantly heightened risk of CAD among SLE patients in European (RR [95% CI] = 2.028 [1.310–3.138], 95% PI: 0.539–7.631; I^2^ = 73.4%, Tau^2^ = 0.1782), East Asian (RR [95% CI] = 2.628 [1.698–4.067], 95% PI: 0.014–487.335; I^2^ = 90.5%, Tau^2^ = 0.1193), and North American populations (RR [95% CI] = 2.711 [2.379–3.089], 95% PI: 2.193–3.352; I^2^ = 0%, Tau^2^ = 0) (Figs. 3B-3D).

#### 3.1.3. Sensitivity analysis and publication bias

Sensitivity analysis was conducted by recalculating the combined RR and associated 95% CI with each study excluded individually, thereby assessing the robustness of the overall findings. This evaluation demonstrated that the exclusion of any single study did not substantially affect the overall conclusion, indicating stability and reliability in the results (Fig. S1). To examine the possibility of publication bias, Begg and Egger tests were applied. The results indicated no significant publication bias in the data analyzed (Begg test: *P* = .584, Egger test: *P* = .080). The funnel plot was presented in Fig. S2.

### 3.2. Mendelian randomization analysis

#### 3.2.1. Selection of instrumental variables

Using a GWAS significance threshold of *P* < 5 × 10^-8^ and removing SNPs exhibiting linkage disequilibrium among individuals of European ancestry, we initially identified 5 SNPs associated with SLE to function as IVs. These SNPs showed no significant links with potential confounders or with the CAD outcome. Following the integration of CAD GWAS data from individuals of European descent and the exclusion of palindromic SNPs with intermediate allele frequencies, these 5 SNPs were employed in MR analysis, with *F*-statistics exceeding 10, suggesting no weak instrument bias. Similarly, 8 SNPs of East Asian ancestry were selected as IVs for the final analysis. Tables S2–S5 provided detailed information on these SNPs.

#### 3.2.2. MR analysis of the causal relationship between SLE and CAD

**Figure 4.**
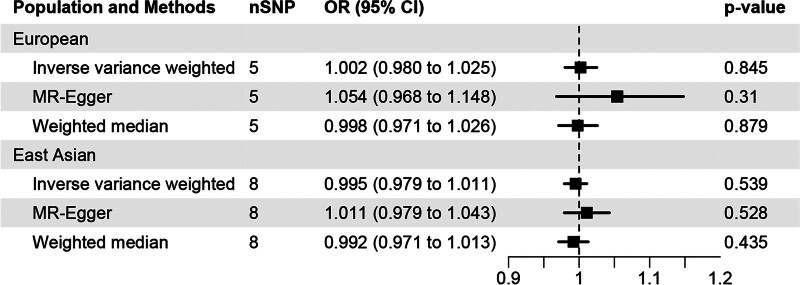
MR analysis of the causal effect of systemic lupus erythematosus on coronary artery disease.

**Figure 5.**
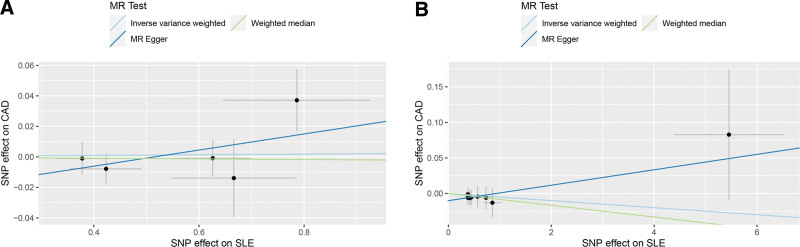
Scatter plots of the results from MR analysis of systemic lupus erythematosus on coronary artery disease. (A) European ancestry; (B) East Asian ancestry.

In this study, we investigated the link between SLE and CAD, with findings illustrated in Figure 4. Through the application of the IVW method, we identified no significant association between SLE and CAD (OR [95% CI] = 1.002 [0.980–1.025], *P* = .845) in European population. This absence of a causal relationship was corroborated by further MR analyses employing both the MR-Egger (OR [95% CI] = 1.054 [0.968–1.148], *P* = .310) and weighted median approach (OR [95% CI] = 0.998 [0.971–1.026], *P* = .879). Consistently, analyses using IVW (OR [95% CI] = 0.995 [0.979–1.011], *P* = .539), MR-Egger (OR [95% CI] = 1.011 [0.979–1.043], *P* = .528), and weighted median (OR [95% CI] = 0.992 [0.971–1.013], *P* = .435) methods also revealed no causal correlation between SLE and CAD in East Asian population. Scatter plots in Figure 5, with MR intercepts approaching zero, suggested minimal horizontal pleiotropy. Furthermore, Fig. S3 provided forest plots showing the estimated causal effects between SLE and CAD.

#### 3.2.3. Sensitivity analyses

The results of the MR sensitivity analysis were comprehensively presented in Table 3. Heterogeneitywas assessed using Cochrane Q statistics, which produced *p*-values exceeding 0.05, suggesting an absence of heterogeneity among the IVs. The MR-Egger regression intercept, employed to assess horizontal pleiotropy, also revealed no significant evidence of pleiotropic effects. These observations are further supported by the MR-PRESSO analysis, which similarly identified no significant outliers indicative of horizontal pleiotropy. Additionally, a leave-one-out analysis, designed to assess the impact of individual SNPs, demonstrated no significant effect on the overall conclusion (Fig. S4). The funnel plots, depicted in Fig. S5, confirmed that there was no significant bias present, thereby reinforcing the reliability of our results.

**Table 3:** Sensitivity analysis of the MR analysis results of the causal association between systemic lupus erythematosus and coronary artery disease.

Population	Exposure/Outcome	Heterogeneity Test		Pleiotropy test		MR-PRESSO
		Cochran Q Test	*p*	Egger Intercept	*p*	Global Test (*p*)
European	SLE/CAD	4.164	0.384	-0.027	0.313	0.432
East Asian	SLE/CAD	2.099	0.954	-0.010	0.302	0.936

CAD = coronary artery disease, SLE = systemic lupus erythematosus.

## 4. Discussion

SLE is often regarded as the archetype of autoimmune rheumatic disorders, as it can present with nearly all clinical features associated with autoimmune conditions.^[43,44]^ In this comprehensive analysis, data from 13 cohort studies were pooled to evaluate the risk of CAD among SLE patients compared to the general or healthy population. The findings indicated that SLE patients face a significantly higher risk of developing CAD. Subgroup analyses revealed that the risk of CAD in SLE patients is more than double that of the general or control population, regardless of whether the population is European, East Asian, or North American. Additionally, MR analysis, utilizing GWAS data from European and East Asian populations, indicated no genetically predicted causal link between SLE and the incidence of CAD in these populations. Sensitivity analyses of both the meta-analysis and MR analysis confirmed the robustness of our findings. Consequently, our results offer valuable insights into the incidence of CAD among SLE patients.

The clinical presentation of CAD encompasses a range of manifestations, from angina and limited functional capacity to acute myocardial infarction and sudden cardiac death.^[45]^ Research consistently indicates that individuals with SLE experience a higher incidence of atherosclerosis and consequently CAD compared to the general population.^[46]^ Atherosclerosis leads to the narrowing of blood vessels due to plaque accumulation, with some plaques being susceptible to rupture, potentially resulting in MI. A 2021 cohort study reported that among more than 4000 SLE patients, the incidence of MI was 9.6 events per 1000 person-years, compared to 4.9 events per 1000 person-years in the control group.^[11]^ The Framingham Offspring Study highlighted that women with SLE aged 35 to 44 faced a risk of MI over 50 times greater than that of similarly aged female participants.^[47]^ Furthermore, SLE patients exhibited a significantly higher incidence of CAD, angina pectoris, and myocardial infarction (MI) compared to controls of the same age group.^[48]^ Our meta-analysis, which consolidated data from previous cohort studies, confirmed that SLE patients are at a significantly elevated risk of CAD across European, East Asian, and North American populations.

The precise mechanisms by which SLE contributes to CAD remain under exploration. Key factors implicated include coronary vasculitis, vasospasm, thrombotic incidents, hypertensive remodeling, and accelerated atherosclerosis.^[49]^ Normally, the vascular system maintains homeostasis by regulating vasomotor responses, hemostatic balance, and inflammatory processes. Endothelial dysfunction, marked by impaired vasodilation and a state conducive to inflammation and thrombosis, plays a critical role in atherosclerosis development and serves as a predictor for future cardiovascular complications.^[50,51]^ Elevated serum concentrations of cell adhesion molecules and various endothelial dysfunction biomarkers have been documented in SLE patients, in comparison to healthy controls, and these elevations correlate with SLE disease activity indicators.^[52,53]^ Numerous studies report that SLE patients, even in the absence of clinically diagnosed CVD, exhibit reduced endothelium-dependent flow-mediated dilation in medium-sized arteries, indicating that endothelial dysfunction precedes cardiovascular events in this population.^[54–56]^ Additionally, in SLE, the pro-inflammatory cytokine environment, alongside elevated oxidative stress, intensifies the oxidation of low-density lipoprotein (LDL). Under typical conditions, high-density lipoprotein (HDL) effectively mitigates this process; however, a subset of HDL particles exhibit pro-inflammatory properties and fail to inhibit LDL oxidation in SLE.^[57]^ The resultant oxidized LDL (oxLDL) stimulates endothelial cells to overexpress adhesion molecules, leading to the recruitment of circulating monocytes/macrophages to the subendothelial area, where they phagocytose oxLDL and other lipids, forming “foam” cells-early indicators of atherosclerotic plaque development.^[57]^ Endothelial dysfunction and arteriosclerosis caused by SLE itself may partly explain the increased risk of CAD in SLE patients.

Our MR analysis did not identify a genetic causal relationship between SLE and CAD. Nevertheless, our meta-analysis of large-scale population data revealed a significantly elevated risk of CAD in individuals with SLE. This elevated risk may be attributed to the higher prevalence of traditional CAD risk factors in individuals with SLE, which could confound the association. For instance, dyslipidemia, a well-known risk factor for CAD, is prevalent among SLE patients. Research found that over one-third of lupus patients had hypercholesterolemia at diagnosis, with this figure rising to over 60% 3 years later.^[58,59]^ The dyslipidemia in SLE follows a distinctive pattern, characterized by elevated very LDL (VLDL) and triglyceride levels, along with reduced HDL levels, which is further exacerbated during active disease.^[60,61]^ Furthermore, factors such as ongoing disease activity, long-term corticosteroid therapy, accumulated disease-related damage, and extended disease duration contribute to the increased CAD risk in SLE.^[62]^ High-dose glucocorticoids are linked to more than a twofold increase in cardiovascular events and exacerbate traditional risk factors like dyslipidemia, hypertension, and hyperglycemia.^[62]^ Although the cohort studies included in our meta-analysis adjusted for confounding variables (e.g., age, gender, diabetes, hypertension, and dyslipidemia) when calculating the RR and 95% CI for CAD risk in SLE patients, it is challenging to eliminate all potential confounders. Additionally, MR relies on known genetic variants associated with SLE, which may not fully capture the disease’s pathophysiological mechanisms, potentially underestimating the impact of SLE on CAD. Therefore, further research is needed to explore the potential mechanisms between SLE and CAD, particularly focusing on possible mediating factors such as inflammatory markers and drug effects.

Several limitations inherent to the MR approach may have impacted our findings. First, the genetic variants used as proxies for SLE may exhibit pleiotropic effects, influencing CAD risk through pathways unrelated to SLE. Additionally, the statistical power of MR can be constrained by the use of weak genetic instruments, particularly for complex traits like SLE. Moreover, MR cannot fully account for unexamined confounders or mediating factors that may contribute to the observed association in meta-analysis. To address these limitations, we performed sensitivity analyses, including assessments for horizontal pleiotropy and heterogeneity, to evaluate the robustness of our findings. These analyses did not reveal significant violations of MR assumptions, supporting the credibility of our conclusions. However, it remains essential to interpret MR findings within the context of its inherent limitations. The meta-analysisand MR approaches each contribute unique strengths to the investigation of the SLE-CAD relationship. Meta-analysis synthesizes evidence from observational studies, capturing real-world associations that may reflect a combination of direct and indirect effects. In contrast, MR provides an alternative framework for causal inference, aiming to determine whether the observed relationship is likely mediated by direct biological pathways. Disparities between the results of these 2 approaches underscore the necessity of employing diverse methodologies to achieve a more comprehensive understanding of complex disease interactions. In this study, the combined application of meta-analysis and MR highlights the importance of caution when interpreting observational findings as causal. While meta-analysis suggests a potential association between SLE and CAD, MR results indicate that this relationship may not be directly causal. Future studies employing complementary methodologies, such as mediation analysis or functional genomics, are warranted to elucidate the biological mechanisms underlying this relationship.

Our study is the first to explore a causal association between SLE and CAD using population- and genetic-level data. Nonetheless, this study faces several limitations. First, the meta-analysis exhibited statistical heterogeneity. Despite this, sensitivity analyses, along with Begg and Egger tests, confirmed the robustness of our findings and indicated no publication bias. Subgroup analyses suggested that racial differences within the study populations may account for the heterogeneity. Second, although RR values from the included studies were adjusted for numerous confounding factors such as demographics, lifestyle, and clinical variables, the impact of commonly used SLE treatments, particularly glucocorticoids, was not considered in all included studies. Long-term glucocorticoid use is associated with atherosclerotic risk factors including hypertension, diabetes, and dyslipidemia, and their exclusion may have significantly influenced the study outcomes. Third, although Egger and Begg tests did not indicate significant publication bias in our meta-analysis, the included studies might over-represent published positive findings. Future meta-analyses can incorporate unpublished data, such as those available in preprint repositories, to provide a more comprehensive assessment of the relationship between SLE and CAD. Fourth, our MR analysis was restricted to participants of European and East Asian ancestry, thereby limiting the generalizability of our findings to other ethnic groups. Fifth, MR methods depend on stringent assumptions regarding the independence of IVs. The extensive pleiotropy associated with complex traits like SLE and CAD complicates these assumptions, potentially biasing analyses by excluding established associations and yielding overly conservative estimates of causal effects and underlying mechanisms. To more definitively determine the causal relationship between SLE and CAD, larger GWAS for SLE are necessary.

## 5. Conclusion

In conclusion, our meta-analysis revealed that SLE patients face a substantially higher risk of CAD compared to the general population or health controls. This elevated risk was also observed among SLE patients across European, East Asian, and North American groups. However, MR analysis did not support a genetic predictive causal relationship between SLE and CAD in populations of European and East Asian ancestry. Considering the potential indirect effects of SLE on CAD risk, along with the risks associated with SLE treatments, clinicians should remain cautious and proactive in monitoring CAD in SLE patients.

## Author contributions

**Conceptualization:** Yan Fan.

**Data curation:** Ping Yuan, Li Fu, Min Ge.

**Formal analysis:** Ping Yuan, Li Fu, Min Ge.

**Investigation:** Ping Yuan, Yan Fan.

**Methodology:** Ping Yuan, Yan Fan.

**Software:** Ping Yuan.

**Supervision:** Yan Fan.

**Writing – original draft:** Ping Yuan.

**Writing – review & editing:** Yan Fan.














