# Supplementary material for: Causal relationship between systemic lupus erythematosus and coronary artery disease: Insights from a meta-analysis and Mendelian randomization
Source: Medicine (Baltimore). 2026 May 15;105(20):e48748. doi: 10.1097/MD.0000000000048748 (PMC13183037; doi:10.1097/MD.0000000000048748)

**Fig. S1** Sensitivity analysis of the causal association between systemic lupus erythematosus and coronary artery disease.


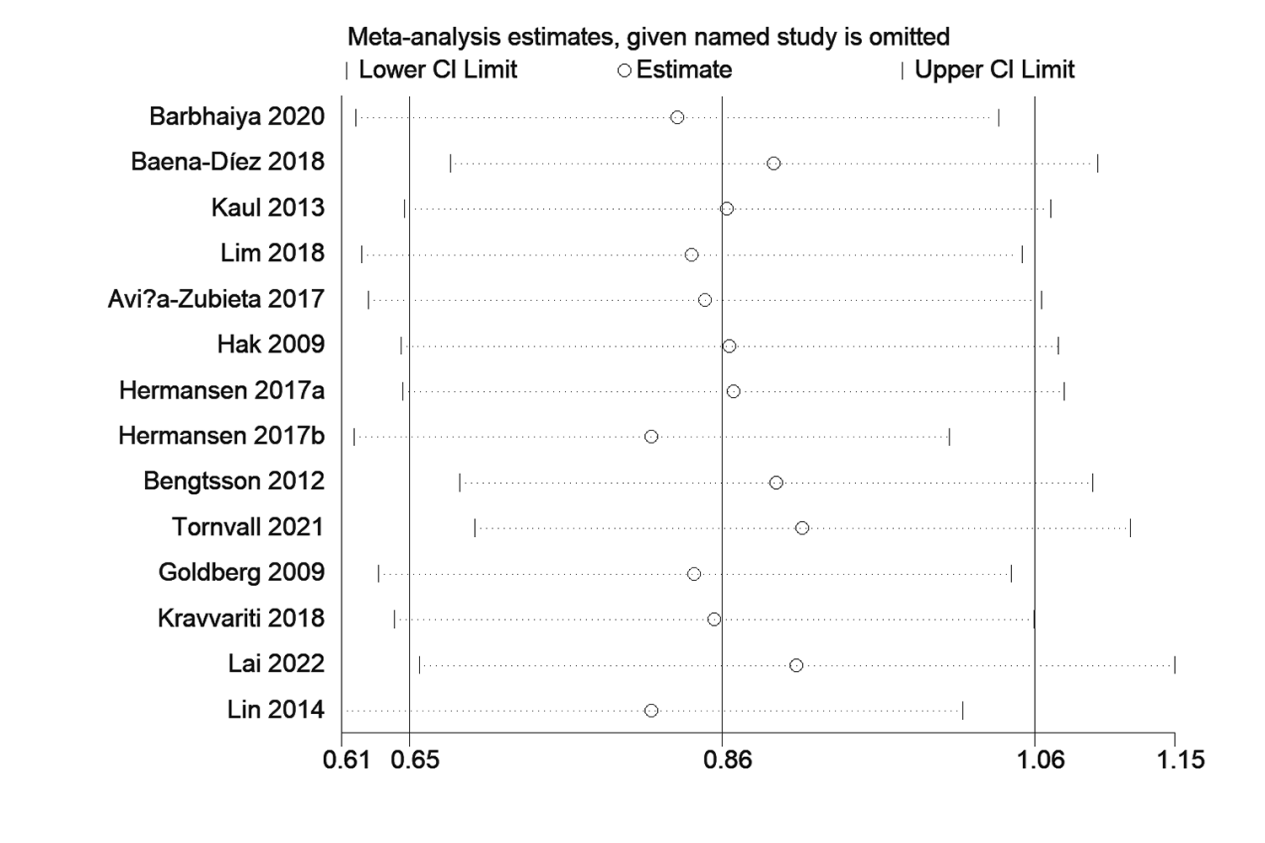


**Fig. S2** Funnel plot of the causal association between systemic lupus erythematosus and coronary artery disease.


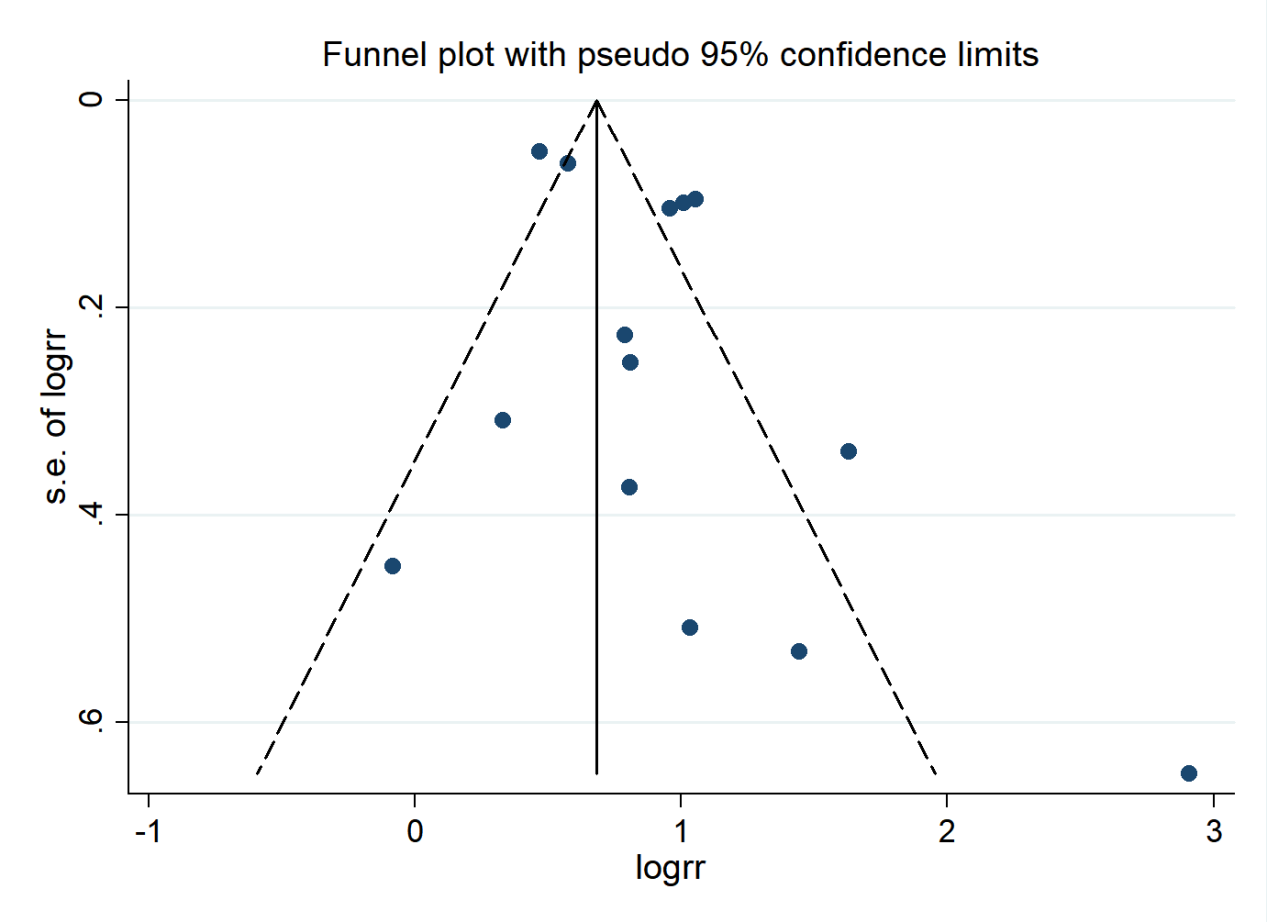


**Fig. S3** Forest plots of the results from MR analysis of systemic lupus erythematosus on coronary artery disease. (A) European population; (B) East Asian population.


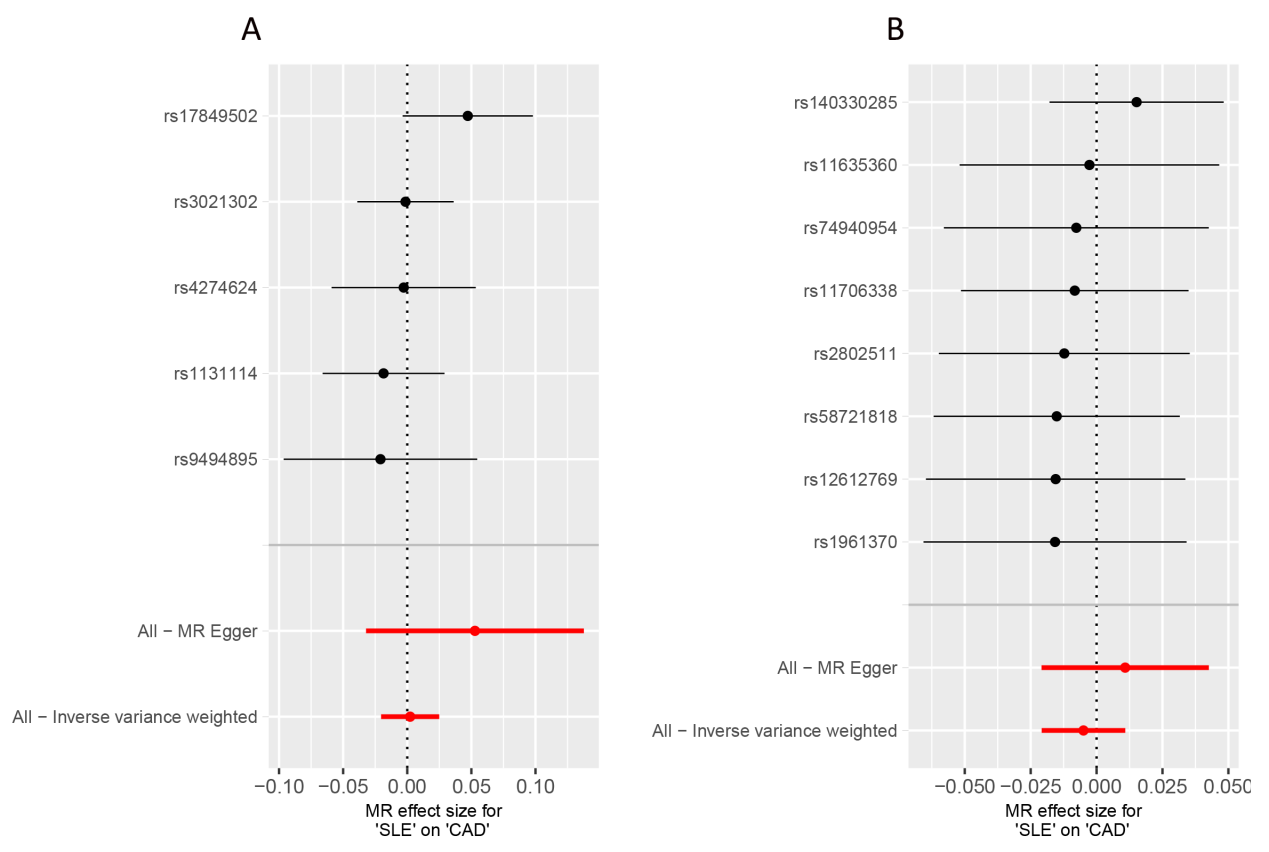


**Fig. S4** Leave-one-out analysis of the results from MR analysis of systemic lupus erythematosus on coronary artery disease. (A) European population; (B) East Asian population.


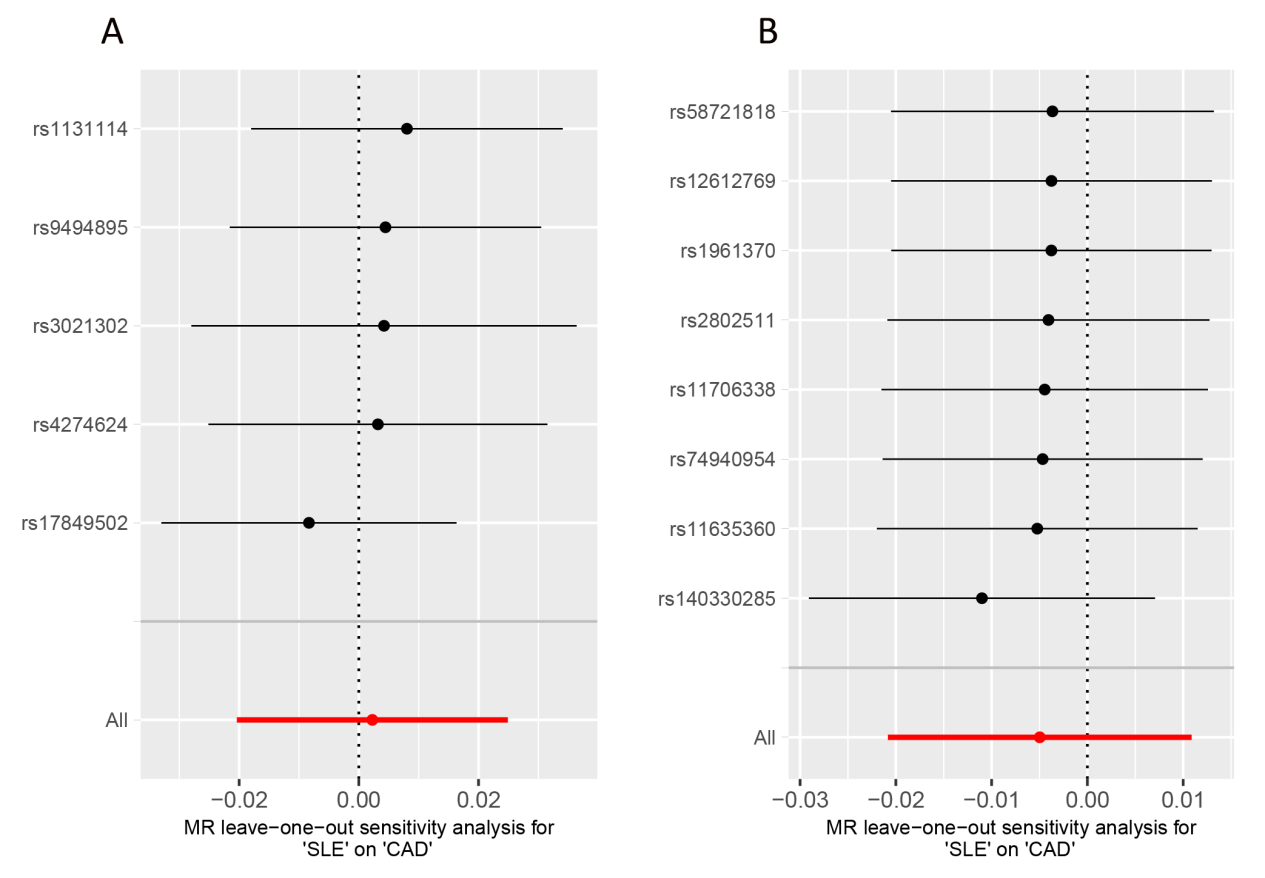


**Fig. S5** Funnel plots of the results from MR analysis of systemic lupus erythematosus on coronary artery disease. (A) European population; (B) East Asian population.


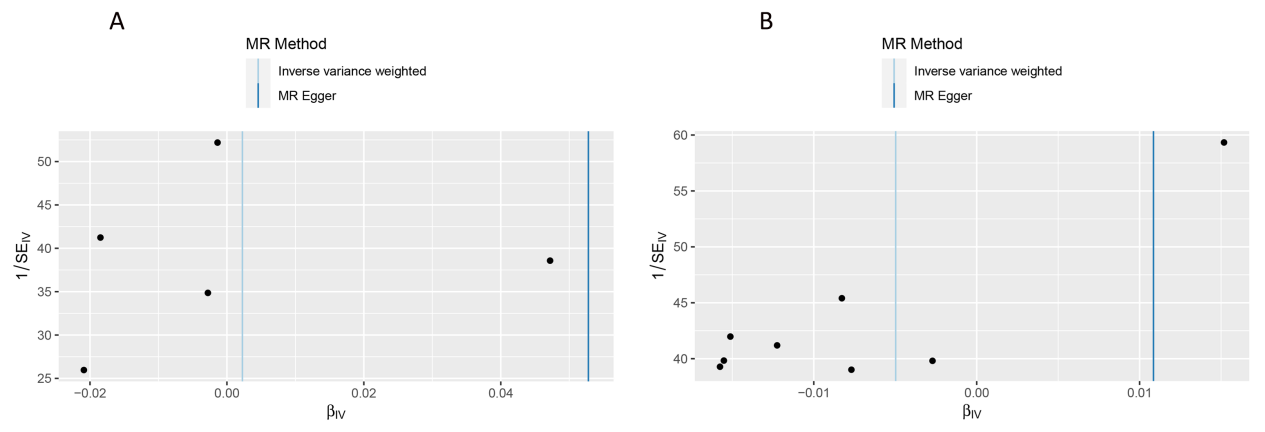

Supplement: Supplementary file 2 [file medi-105-e48748-s002.docx]
