# Supplementary material for: Causal relationship between systemic lupus erythematosus and coronary artery disease: Insights from a meta-analysis and Mendelian randomization
Source: Medicine (Baltimore). 2026 May 15;105(20):e48748. doi: 10.1097/MD.0000000000048748 (PMC13183037; doi:10.1097/MD.0000000000048748)
Supplement: Supplementary file 3 [file medi-105-e48748-s003.doc]

| Table S2. The characteristic of instrumental variables for the exposure of systemic lupus erythematosus in European. | | | | | | | | | |
| --- | --- | --- | --- | --- | --- | --- | --- | --- | --- |
| SNP | Effect_allele | Other_allele | EAF | Beta | SE | p-val | R^2^ | *F* | Note |
| rs17849502 | T | G | 0.048294 | 0.7862 | 0.14 | 1.95E-08 | 6.53002E-05 | 31.53611633 |  |
| rs4274624 | T | C | 0.74417 | -0.3778 | 0.0529 | 9.37E-13 | 0.000105609 | 51.00476659 |  |
| rs3021302 | C | T | 0.141593 | 0.6262 | 0.0724 | 5.25E-18 | 0.000154887 | 74.80785385 |  |
| rs9494895 | T | C | 0.0398049 | 0.6663 | 0.1193 | 2.31E-08 | 6.45897E-05 | 31.1929853 |  |
| rs1131114 | C | T | 0.179164 | 0.4233 | 0.0678 | 4.36E-10 | 8.07114E-05 | 38.97941801 |  |
| SNP, single nucleotide polymorphism; EAF, effect allele frequency; SE, standard error; p-val, p-value; F, F-statistic. | | | | | | | | | |
