# Supplementary material for: Causal relationship between systemic lupus erythematosus and coronary artery disease: Insights from a meta-analysis and Mendelian randomization
Source: Medicine (Baltimore). 2026 May 15;105(20):e48748. doi: 10.1097/MD.0000000000048748 (PMC13183037; doi:10.1097/MD.0000000000048748)
Supplement: Supplementary file 4 [file medi-105-e48748-s004.docx]

**PubMed 747**

#1 (systemic lupus erythematosus) OR (lupus erythematosus, systemic) OR (lupus erythematosus disseminatus) OR (libman sacks disease) OR (libman-sacks disease) OR (disease, libman-sacks)

#2 (coronary artery disease) OR (disease, coronary artery) OR (artery disease, coronary) OR (coronary arteriosclerosis) OR (coronary arterioscleroses) OR (coronary atherosclerosis) OR (coronary atheroscleroses)

#3 #1 AND #2

**Web of Science 1587**

#1 TS=((systemic lupus erythematosus) OR (lupus erythematosus, systemic) OR (lupus erythematosus disseminatus) OR (libman sacks disease) OR (libman-sacks disease) OR (disease, libman-sacks))

#2 TS=((coronary artery disease) OR (disease, coronary artery) OR (artery disease, coronary) OR (coronary arteriosclerosis) OR (coronary arterioscleroses) OR (coronary atherosclerosis) OR (coronary atheroscleroses))

#3 #1 AND #2

**Embase 697**

#1 'systemic lupus erythematosus':ti,ab,kw OR 'lupus erythematosus, systemic':ti,ab,kw OR 'lupus erythematosus disseminatus':ti,ab,kw OR 'libman sacks disease':ti,ab,kw OR 'libman-sacks disease':ti,ab,kw OR 'disease, libman-sacks':ti,ab,kw

#2 'coronary artery disease':ti,ab,kw OR 'disease, coronary artery':ti,ab,kw OR 'artery disease, coronary':ti,ab,kw OR 'coronary arteriosclerosis':ti,ab,kw OR 'coronary arterioscleroses':ti,ab,kw OR 'coronary atherosclerosis':ti,ab,kw OR 'coronary atheroscleroses':ti,ab,kw

#3 #1 AND #2

**The Cochrane Library 60**

#1 All Text=((systemic lupus erythematosus) OR (lupus erythematosus, systemic) OR (lupus erythematosus disseminatus) OR (libman sacks disease) OR (libman-sacks disease) OR (disease, libman-sacks))

#2 All Text=((coronary artery disease) OR (disease, coronary artery) OR (artery disease, coronary) OR (coronary arteriosclerosis) OR (coronary arterioscleroses) OR (coronary atherosclerosis) OR (coronary atheroscleroses))

#3 #1 AND #2
