# Supplementary material for: Causal relationship between systemic lupus erythematosus and coronary artery disease: Insights from a meta-analysis and Mendelian randomization
Source: Medicine (Baltimore). 2026 May 15;105(20):e48748. doi: 10.1097/MD.0000000000048748 (PMC13183037; doi:10.1097/MD.0000000000048748)
Supplement: Supplementary file 5 [file medi-105-e48748-s005.doc]

| **Table S4.** The characteristic of instrumental variables for the exposure of systemic lupus erythematosus in East Asian. | | | | | | | | | |
| --- | --- | --- | --- | --- | --- | --- | --- | --- | --- |
| SNP | Effect_allele | Other_allele | EAF | Beta | SE | *p*-val | R^2^ | *F* | Note |
| rs12612769 | C | A | 0.298590103 | 0.418811298 | 0.088874245 | 2.45E-06 | 0.000125977 | 22.20649407 |  |
| rs11706338 | G | T | 0.09433705 | 0.732723222 | 0.145563122 | 4.81E-07 | 0.000143739 | 25.33799578 |  |
| rs74940954 | T | C | 0.160759394 | 0.568036961 | 0.116502169 | 1.08E-06 | 0.000134861 | 23.77279456 |  |
| rs138408489 | A | G | 0.011844938 | 2.20324317 | 0.482313128 | 4.92E-06 | 0.000118379 | 20.86708433 |  |
| rs140330285 | G | A | 0.003419797 | 5.453964388 | 1.065004347 | 3.04E-07 | 0.000148771 | 26.22508511 |  |
| rs58721818 | T | C | 0.059679046 | 0.8563161 | 0.178204526 | 1.55E-06 | 0.000130989 | 23.09010102 |  |
| rs1961370 | C | A | 0.418229029 | 0.378361562 | 0.081471319 | 3.42E-06 | 0.000122352 | 21.56748977 |  |
| rs2802511 | G | A | 0.262946423 | 0.4433832 | 0.091759468 | 1.35E-06 | 0.000132453 | 23.34811168 |  |
| rs11635360 | G | A | 0.560393122 | 0.379899945 | 0.080489083 | 2.36E-06 | 0.000126378 | 22.27714745 |  |
| SNP, single nucleotide polymorphism; EAF, effect allele frequency; SE, standard error; *p*-val, *p*-value; *F*, *F*-statistic. | | | | | | | | | |
