# Supplementary material for: Causal relationship between systemic lupus erythematosus and coronary artery disease: Insights from a meta-analysis and Mendelian randomization
Source: Medicine (Baltimore). 2026 May 15;105(20):e48748. doi: 10.1097/MD.0000000000048748 (PMC13183037; doi:10.1097/MD.0000000000048748)
Supplement: Supplementary file 7 [file medi-105-e48748-s007.doc]

| **Table S5.** The characteristic of instrumental variables for the exposure of systemic lupus erythematosus and the outcome of coronary artery disease in East Asian. | | | | | | | | | | | | | | | |
| --- | --- | --- | --- | --- | --- | --- | --- | --- | --- | --- | --- | --- | --- | --- | --- |
| SNP | Effect_allele.exposure | Other_allele.exposure | Effect_allele.outcome | Other_allele.outcome | Beta.exposure | Beta.outcome | EAF.exposure | EAF.outcome | Palindromic | Ambiguous | SE.outcome | *p*-val.outcome | SE.exposure | *p*-val.exposure | Note |
| rs11635360 | G | A | G | A | 0.379899945 | -0.00103077 | 0.560393122 | 0.562624 | FALSE | FALSE | 0.0095431 | 0.913986 | 0.080489083 | 2.36E-06 |  |
| rs11706338 | G | T | G | T | 0.732723222 | -0.00606437 | 0.09433705 | 0.0943021 | FALSE | FALSE | 0.0161374 | 0.707068 | 0.145563122 | 4.81E-07 |  |
| rs12612769 | C | A | C | A | 0.418811298 | -0.0064998 | 0.298590103 | 0.302894 | FALSE | FALSE | 0.0105147 | 0.536466 | 0.088874245 | 2.45E-06 |  |
| rs140330285 | G | A | G | A | 5.453964388 | 0.0827984 | 0.003419797 | 0.00329953 | FALSE | FALSE | 0.091916 | 0.367692 | 1.065004347 | 3.04E-07 |  |
| rs1961370 | C | A | C | A | 0.378361562 | -0.00596239 | 0.418229029 | 0.4192 | FALSE | FALSE | 0.00963255 | 0.535927 | 0.081471319 | 3.42E-06 |  |
| rs2802511 | G | A | G | A | 0.4433832 | -0.00543176 | 0.262946423 | 0.263912 | FALSE | FALSE | 0.0107645 | 0.613841 | 0.091759468 | 1.35E-06 |  |
| rs58721818 | T | C | T | C | 0.8563161 | -0.0129477 | 0.059679046 | 0.0598405 | FALSE | FALSE | 0.0203996 | 0.525623 | 0.178204526 | 1.55E-06 |  |
| rs74940954 | T | C | T | C | 0.568036961 | -0.00436893 | 0.160759394 | 0.14923 | FALSE | FALSE | 0.0145602 | 0.764131 | 0.116502169 | 1.08E-06 |  |
| SNP, single nucleotide polymorphism; EAF, effect allele frequency; SE, standard error; *p*-val, *p*-value. | | | | | | | | | | | | | | | |
